# Decompressive Pathology in Cetaceans Based on an Experimental Pathological Model

**DOI:** 10.3389/fvets.2021.676499

**Published:** 2021-06-08

**Authors:** Alicia Velázquez-Wallraf, Antonio Fernández, Maria José Caballero, Andreas Møllerløkken, Paul D. Jepson, Marisa Andrada, Yara Bernaldo de Quirós

**Affiliations:** ^1^Veterinary Histology and Pathology, Atlantic Center for Cetacean Research, University Institute of Animal Health and Food Safety (IUSA), Veterinary School, University of Las Palmas de Gran Canaria, Canary Islands, Spain; ^2^Faculty of Engineering, Norwegian University of Science and Technology, NTNU, Trondheim, Norway; ^3^Institute of Zoology, Zoological Society of London, London, United Kingdom

**Keywords:** gas bubble, stranded cetaceans, pathology, rabbit model, decompression sickness

## Abstract

Decompression sickness (DCS) is a widely known clinical syndrome in human medicine, mainly in divers, related to the formation of intravascular and extravascular gas bubbles. Gas embolism and decompression-like sickness have also been described in wild animals, such as cetaceans. It was hypothesized that adaptations to the marine environment protected them from DCS, but in 2003, decompression-like sickness was described for the first time in beaked whales, challenging this dogma. Since then, several episodes of mass strandings of beaked whales coincidental in time and space with naval maneuvers have been recorded and diagnosed with DCS. The diagnosis of human DCS is based on the presence of clinical symptoms and the detection of gas embolism by ultrasound, but in cetaceans, the diagnosis is limited to forensic investigations. For this reason, it is necessary to resort to experimental animal models to support the pathological diagnosis of DCS in cetaceans. The objective of this study is to validate the pathological results of cetaceans through an experimental rabbit model wherein a complete and detailed histopathological analysis was performed. Gross and histopathological results were very similar in the experimental animal model compared to stranded cetaceans with DCS, with the presence of gas embolism systemically distributed as well as emphysema and hemorrhages as primary lesions in different organs. The experimental data reinforces the pathological findings found in cetaceans with DCS as well as the hypothesis that individuality plays an essential role in DCS, as it has previously been proposed in animal models and human diving medicine.

## Introduction

Decompression sickness (DCS) is a widely known clinical syndrome in human medicine, mainly in recreational and professional divers. It is considered to occur when the sum of the gases dissolved in the tissues exceeds the environmental pressure, causing the formation of intravascular and extravascular gas bubbles. The presence of gas embolism as noted by ultrasound must be observed for its confirmatory clinical diagnosis. In these cases, the patient is treated with a hyperbaric chamber. If the gas and the symptoms resolve, the diagnosis of DCS is definitive (1). In forensic investigations, gas bubble-related lesions are the main findings (2, 3). These bubbles can result in mechanical, biochemical, and embolic damage with different severity levels depending on their number and their size (1). The respiratory system is the most affected organ by DCS when the amount of bubbles exceeds the pulmonary capillaries' capacity to eliminate them, resulting in severe lung damage (4).

Cetaceans are mammals that returned to the marine environment 60 million years ago and have developed behavioral, anatomical, and physiological adaptations for this new habitat, including those related to diving (5). It was hypothesized that these adaptations protected them from possible DCS, but in 2003, lesions compatible with DCS were described for the first time in beaked whales stranded coincidentally in time and space with naval exercises using high-intensity and mid-frequency active sonars (6, 7). This first description of a decompression-like sickness in beaked whales broke the dogma that cetaceans were immune to this disease (7). These findings have also been found in other beaked whale (BW) strandings associated with naval maneuvers (8–11) as well as in Risso's dolphins but, in this case, were caused naturally due to an interaction with their preys during feeding (12). Gas embolism and decompression-like sickness have also been described in other wild animals such as sea turtles (13).

In cetaceans, the diagnosis of DCS is limited to forensic investigations and its pathological gas bubble-associated lesions since a clinical diagnosis is not possible due to obvious logistical and ethical restrictions. For this reason, it is necessary to resort to experimental animal models to contrast the macroscopic and microscopic lesions and to support the pathological diagnosis described in marine mammals affected by DCS. To our knowledge, there are very few publications focused on the pathological study of DCS in humans or other species (14–17), and almost all the articles on animal experimentation focus on the analysis of specific tissues for the application of preventive treatments in DCS (4, 18–20). Furthermore, there is no pathological comparative study showing gross and histological findings in experimental and natural DCS. Therefore, the objective of this study is to validate the pathological results of cetaceans through an experimental model wherein a complete and detailed histopathological analysis is performed. For this purpose, an experimental rabbit model was performed in which severe DCS is reproduced, presenting the pathological results in these animals and then comparing them with the pathological findings in cetaceans.

## Materials and Methods

For this study, 18 males of New Zealand white rabbits of 3.15 ± 0.65 kg were used. These animals were divided into compression/decompression model (C/D) (*n* = 14) and control group (C) (*n* = 4).

All experiments were accomplished following the European Union's laboratory animals' regulation and were conducted under surgical anesthesia: subcutaneous injections of medetomidine (0.5 mg/kg) and ketamine (25 mg/kg). The C/D model was carried out in the experimental animal facilities of St. Olav University Hospital NTNU, Norway (Trondheim, Norway), and the Norwegian Committee for Animal Experiments approved the protocol (2154). The control group was carried out in the experimental animal facilities of Dr. Negrín University Hospital (Las Palmas de Gran Canaria, Spain), and the Ethical Committee for Animal Experiments of the University of Las Palmas de Gran Canaria approved the protocol (CEEBA-HUGCDN 002/2010).

### The Compression/Decompression Model

The rabbits were anesthetized with the protocol described above and compressed in pairs in a dry, hyperbaric chamber (Animal Chamber System, NUT, Haugesund, Norway) with a diving profile selected to induce severe decompression stress with excessive amounts of intra-corporal gas formation: eight absolute atmospheres during 45 min, followed by fast decompression (0.33 m/s) to one atmosphere (21). One animal appeared dead when recovered from the chamber, and it remains unclear at what time during the treatment the animal died; thus, it was withdrawn from the study. The animals were monitored for 1 h after decompression. A group of animals (*n* = 8) died within 25 min post-decompression (C/D mortality group), while the rest (*n* = 5) survived the observation period of 1 h and were euthanized with an intraperitoneal injection of diluted pentobarbital (200 mg/kg) (C/D euthanized group).

### Control Group

The rabbits were first anesthetized with the protocol described above and later euthanized with an intraperitoneal injection of diluted pentobarbital (200 mg/kg) as in the C/D euthanized group.

### Pathological Study

Necropsy was carried out for each rabbit in a dorsal decubitus position. Dissection was carefully done to avoid severing large blood vessels following the method of Bernaldo de Quirós et al. (22) to characterize the presence of intravascular and extravascular gas bubbles using a gas score index. This index-based method consists of giving a gas score from 0 to 6 for each of the defined vascular locations (i.e., subcutaneous veins, femoral veins, mesenteric veins, caudal vena cava, coronary veins, and to the right atrium) and a gas score from 0 to 3 to describe the presence and distribution of extravascular gas (i.e., subcapsular and interstitial emphysema) that may affect different organs. The sum of the gas score of each intravascular and extravascular location calculates the total gas score in each rabbit. In the current study, the mode of each group for intra- and extravascular locations has been calculated.

Representative samples of the lung, trachea, superficial cervical lymph node, spleen, central nervous system, heart, liver, stomach, small and large intestine, mesenteric lymph node, kidney, urinary bladder, and skeletal muscle (gastrocnemius) were collected and fixed in 10% buffered formalin. These tissues were processed routinely and embedded in paraffin wax, and 5-μm-thick sections were cut and stained with hematoxylin and eosin (23) for microscopic analysis. Histological sections from the heart and skeletal muscle were also stained with phosphotungstic acid hematoxylin and Masson's trichrome (23), respectively, to evidence changes in skeletal and cardiac musculature.

### Comparison With Cetacean Decompressive Pathology

The histopathological results from the animal model were compared with the necropsy reports and histology slides from stranded cetaceans that have been studied and diagnosed with DCS by our research group. This included 31 animals: eight Cuvier's BWs (*Ziphius cavirostris*), one Blainville's BW (*Mesoplodon densirostris*), and one Gervais's BW (*Mesoplodon europaeus*) stranded in the islands of Fuerteventura and Lanzarote (Spain) in 2002 (7); four Cuvier's BWs stranded on these same islands in 2004 (8); four Cuvier's BWs stranded in 2006 and one Cuvier's BWs stranded in 2011, both in Almeria (Spain) (9, 10); 10 Cuvier's BW mass stranding in Corfú (Greece) in 2011 (11), all of them coincidental in time and space with naval exercises; and two Risso's dolphins (*Grampus griseus*) that were diagnosed with a decompressive disease after an interaction with a prey (12). Since the pathological results of these animals have already been published, the comparison with the original pathological results from this study will be addressed in the Discussion section.

## Results

### Presence, Distribution, and Amount of Bubbles

**Figure 1:**
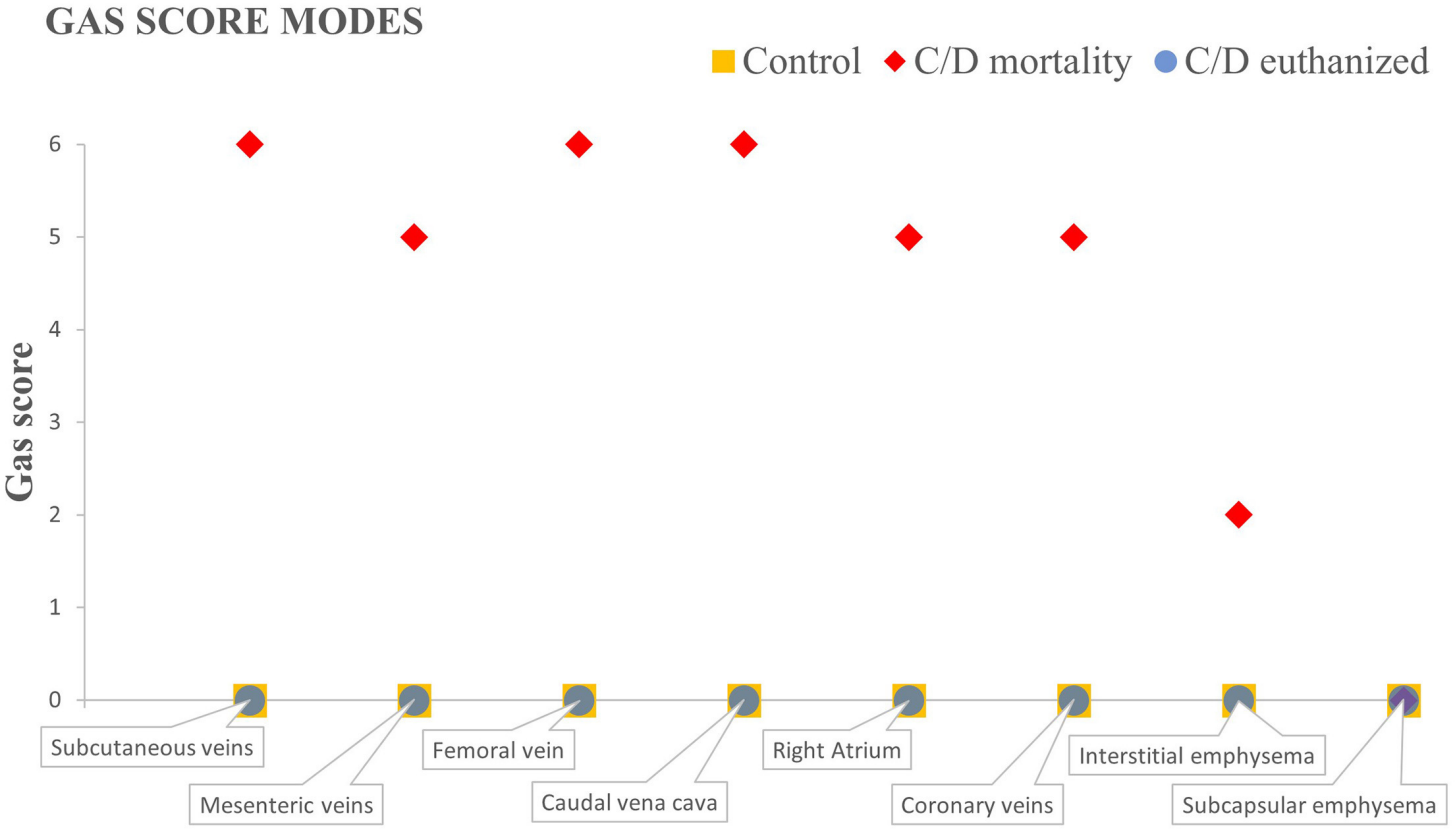
Gas score modes of C/D mortality animals, C/D euthanized animals, and control group in the different locations selected.

Rabbits from the control group presented very few or an absence of gas bubbles. One animal presented few gas bubbles in the mesenteric veins, occasional bubbles in the subcutaneous veins, and scarce bubbles in the adipose tissue (total gas score: 4). One more animal presented only occasional gas bubbles in the mesenteric veins (total gas score: 1). These results were previously reported by Bernaldo de Quirós et al. (22). The resulting gas score mode calculated in this study for all locations was 0 (Figure 1) since the two remaining animals showed no bubbles.

**Figure 2:**
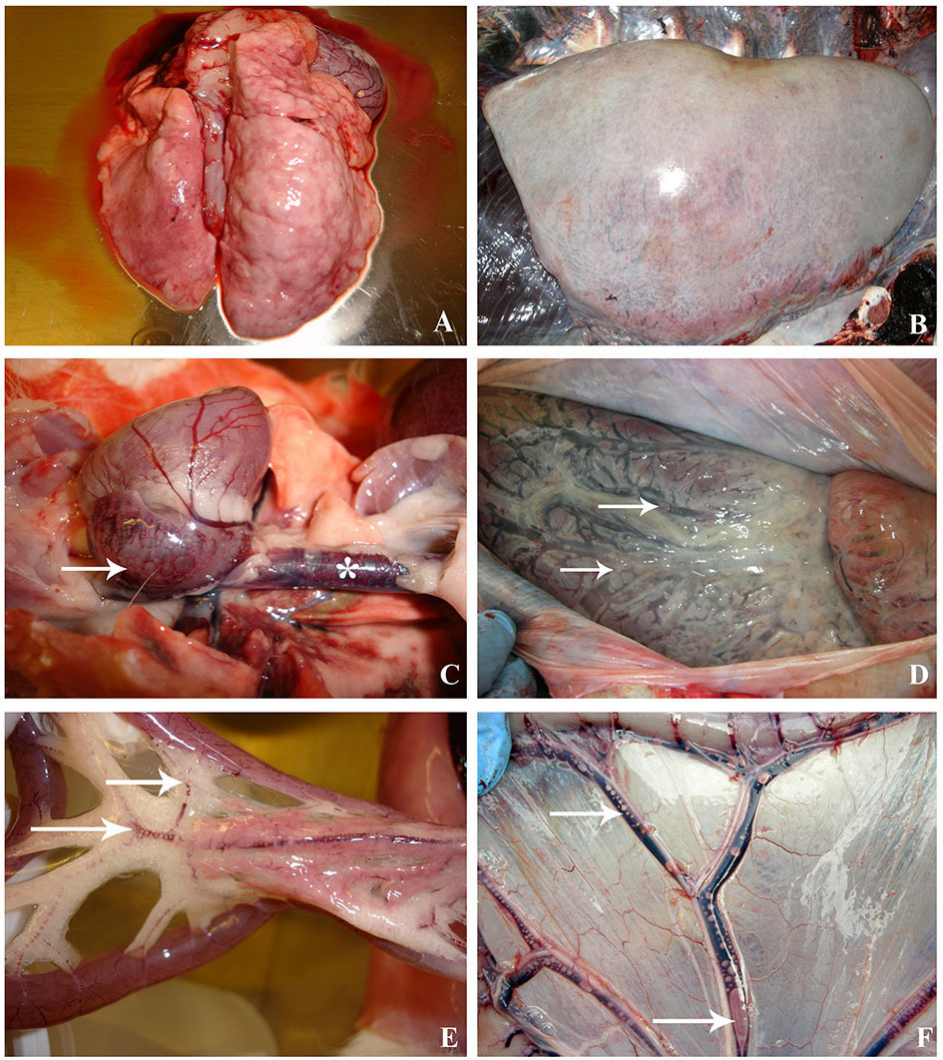
Macroscopic lesions compared between decompression sickness in rabbits (left row) and cetaceans (right row). **(A)** Presence of pale lungs with distended and enlarged areas, mainly denoted in the right side, in a rabbit dead by C/D protocol. **(B)** Emphysematous lungs in a beaked whale diagnosed with decompression-like sickness. **(C)** Heart of a C/D mortality rabbit with congestion and macroscopic bubbles in the right atrium (white arrow) and caudal vena cava (white star). **(D)** Heart of a beaked whale with dilated right atrium due to the presence of macroscopic bubbles, which are also observed in the coronary vessels (white arrows). **(E)** Mesenteric area of a C/D mortality rabbit. Emphysematous fat and congestion of blood vessels running through mesenteric fat. The presence of gas bubbles in the mesenteric veins is denoted (white arrow). **(F)** Mesenteric area of a Risso's dolphin with visible bubbles circulating in the mesenteric vessels (white arrows) and congestion.

In rabbits from the C/D mortality group, gas bubbles were observed in abundant numbers and/or filling complete vessel sections in the subcutaneous veins, the femoral veins, the mesenteric veins (Figure 2E), the caudal vena cava, the right atrium (Figure 2C), and the coronary veins. In addition, a sparse or moderate presence of subcapsular and interstitial emphysema was observed. The gas score mode in the subcutaneous veins, the femoral vein, and the caudal vena cava was 6, while the mesenteric and the coronary veins, along with the right atrium, had a gas score mode of 5 (Figure 1). The gas score mode for interstitial emphysema in this group was 2, while that of subcapsular emphysema was 0. The total gas score ranged from 29 to 40 (22). Additionally, large amounts of gas bubbles were found disseminated through other vascular locations.

Gas bubbles were not found in the C/D euthanized group, with a total gas score of 0 in all animals and a gas score mode for all locations of 0 (22).

### Gross Examination and Histopathology

#### Control Group

Gross findings in this group showed congestion in different organs, such as lung (3/4, 75%), liver (3/4, 75%), kidney (3/4, 75%), spleen (2/4, 50%), and brain (1/4, 25%), and mild multifocal petechial hemorrhages in thymus (3/4, 75%). These findings were confirmed histologically. No other histopathological findings were observed, except hypereosinophilia (2/4, 50%) and vacuolization (2/4, 50%) in muscular cardiac fibers and vacuolization of hepatocytes (1/4, 25%).

#### C/D Model: Mortality Group

**Figure 3:**
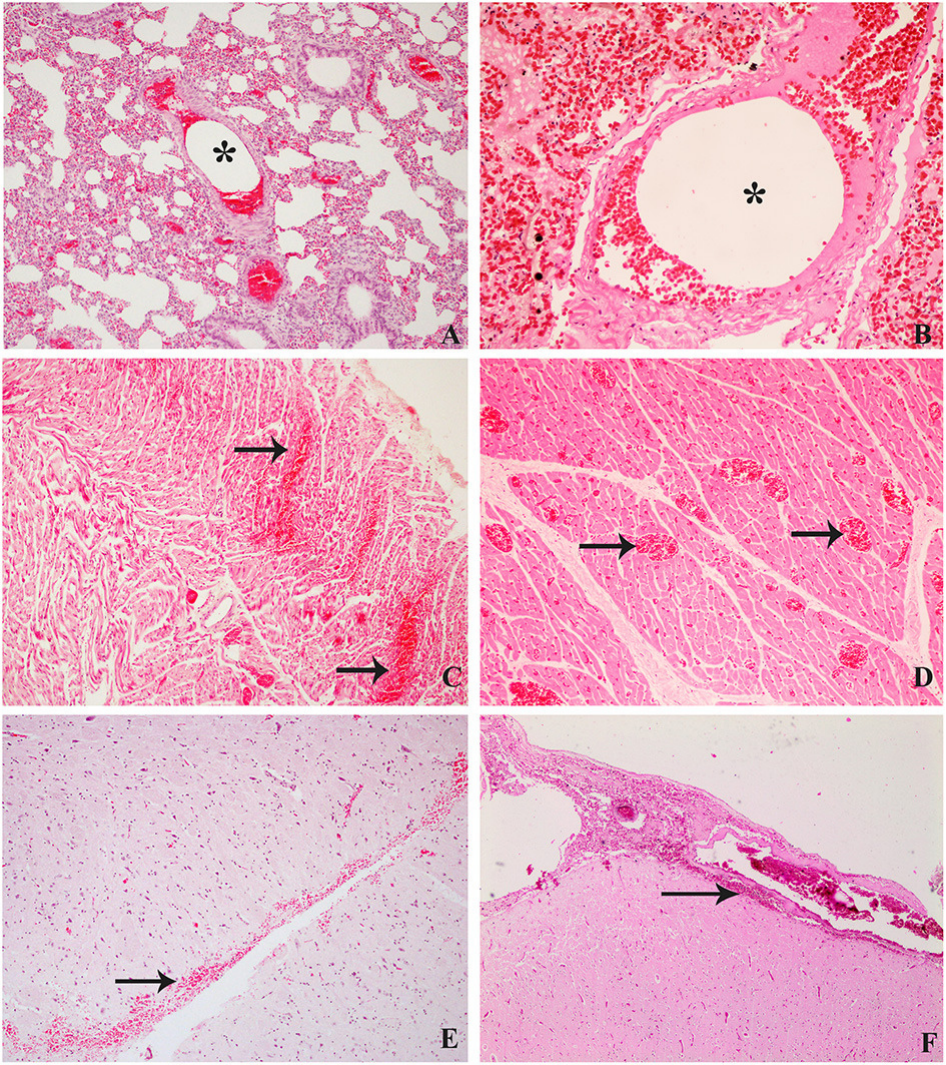
Histological findings compared between decompression sickness in rabbits (left row) and cetaceans (right row) stained routinely with hematoxylin–eosin. **(A)** C/D mortality rabbit. Intravascular bubble-like round empty space among blood cells (black star), mild emphysema, and congestion in pulmonary areas. **(B)** Risso's dolphin, ×10. Microscopic bubble-like cavities circulating within a pulmonary blood vessel (black star). **(C)** C/D mortality rabbit, ×20. Multifocal hemorrhages (black arrows) and congestion of cardiac capillaries. **(D)** Risso's Dolphin, ×10. Presence of congestive capillaries in cardiac tissue (black arrows). **(E)** C/D mortality rabbit, ×20. Hemorrhages in the subarachnoid area of the central nervous system. **(F)** Beaked whale, ×10. Subarachnoid hemorrhages in the central nervous system.

Emphysema was the predominant lesion observed in the lung of C/D mortality animals (6/8, 75%), with grossly voluminous, pale, and gas-distended pulmonary areas (Figure 2A). Other lung findings were congestion (3/8, 38%) and alveolar edema evidenced by exudation of fluid from the cut surface (2/8, 25%). The lung's microscopic appearance showed mild to severe emphysema in all animals (8/8, 100%). Besides these, mild pulmonary congestion (7/8, 88%) and alveolar hemorrhages ranging from mild focal hemorrhages to severe multifocal hemorrhages (4/8, 50%) as well as microscopic intravascular bubble-like round empty spaces surrounded by blood cells (3/8, 38%) were observed (Figure 3A).

Marked subcapsular splenic emphysema was observed (3/8, 38%). Histological emphysema was confirmed in six out of eight animals (75%). Gross cavities underneath the splenic capsule and within splenic parenchyma were observed microscopically along with mild splenic congestion (7/8, 88%). Cerebral congestion was seen in four cases of C/D mortality animals (4/8, 50%), and two animals showed, microscopically, mild local to extensive hemorrhages in the meningeal area and around cerebral capillaries (2/8, 25%) (Figure 3E).

The most relevant finding in the heart was the presence of hemorrhages, mainly in the right ventricle, in the group of C/D mortality animals (3/8, 38%). Mild hemorrhages were confirmed microscopically (4/8, 50%) (Figure 3C), while two showed bubble-like cavities in cardiac capillaries (2/8, 25%). Acute changes such as mild hyaline changes of muscle fibers as well as hypereosinophilia and mild intracytoplasmic vacuolization of injured cardiomyocytes were observed in seven animals out of eight (88%). In contrast, contraction band fibers were detected in only one animal (1/8, 13%). Cardiac muscle fibers were separated by expanded interstitial spaces filled with pale pink material, indicating mild interstitial edema (3/8, 38%).

In the liver of C/D mortality animals, mild hepatomegaly and general congestion were the main gross findings (5/8, 63%). Histologically, moderate congestion was confirmed by the distention of central veins and sinusoids. Mild vacuolization of centrilobular hepatocytes with cytoplasmic ballooning of these cells was also observed (4/8, 50%). Renal congestion was also found in this group (4/8, 50%). Microscopic vascular bubble-like cavities were also observed (2/8, 25%).

Other findings in this group were emphysema (7/8, 88%) and vascular congestion (4/8, 50%) associated with the adipose tissue. At the microscopic analysis of this group's skeletal muscle, acute changes such as mild hypereosinophilia were found in seven animals out of eight (88%) as well as interfibrillar mild interstitial edema (4/8, 50%).

#### C/D Model: Euthanized Group

The lung of C/D euthanized animals showed emphysema (2/5, 40%), while edema was present in one animal of five (20%). Histologically, 100% of animals presented mild lung emphysema, mild congestion (3/5, 60%), and multifocal alveolar hemorrhages (1/5, 20%). While splenic subcapsular emphysema was only macroscopically observed in one animal (1/5, 20%) and congestion in two animals (2/5, 40%), the microscopic analysis revealed mild splenic congestion in 100% of animals (5/5, 100%) and subcapsular and parenchymal emphysema in 80% of animals (4/5). In the brain, gross and microscopic congestion was found (2/5, 40%).

The heart showed no gross lesions in this group, whereas acute cardiomyocyte changes, intracytoplasmic vacuolization, and hypereosinophilia were detected microscopically in 100% of animals (5/5). Mild congestion (2/5, 40%), mild hemorrhages (2/5, 40%) as well as mild interstitial edema (1/5, 20%) were also observed in this group.

Hepatic congestion was present in four animals out of five (80%) in this group and mild congestion (5/5, 100%) with mild hepatocytic vacuolization (3/5, 60%). Renal congestion was observed in 40% (2/5), while congestion was observed in 100% (5/5), with intravascular bubble-like cavities in 60% of the animals (3/5).

Other findings in this group were congestion of the adipose tissue (2/5, 40%) and muscle fiber hypereosinophilia as well as wavy fibers in two animals (2/5, 40%).

#### Comparative Results

As shown in Figure 1, where the different gas score modes of each group are represented, both the control and the C/D euthanized animals do not present macroscopic bubbles in any defined location for the gas score. The C/D mortality group presents bubbles in all locations, varying between abundant number of bubbles and completely filling vessel sections, except the presence of few bubbles leading to interstitial emphysema and the absence of subcapsular emphysema.

As shown in Tables 1, 2, the presence of microscopic bubble-like cavities was another finding also observed with greater incidence in the group of C/D mortality, with these bubbles observed in capillaries and small-sized blood vessels of the lung (38%), the heart (25%), and the kidney (25%). In the C/D euthanized group, the presence of these microscopic bubble-like cavi ties was lower in kidney, although with a relevant percentage (60%).

**Table 1:** Macroscopic findings in each group of rabbits and organs.

	**Lung**				**Heart**	**Thymus**	**Liver**	**Kidney**	**Spleen**		**Brain**	**Fat**	
	**Congestion**	**Edema**	**Emphysema**	**Pneumonia**	**Hemorrhages**	**Petechiae**	**Congestion**	**Congestion**	**Congestion**	**Emphysema**	**Congestion**	**Congestion**	**Emphysema**
C/DMortality	3/8 (38%)	2/8 (25%)	6/8 (75%)	1/8 (13%)	3/8(38%)	0/8(0%)	5/8 (63%)	4/8 (50%)	1/8 (13%)	3/8 (38%)	4/8 (50%)	4/8 (50%)	7/8 (88%)
C/DEuthanized	0/5 (0%)	1/5 (20%)	2/5 (40%)	0/5 (0%)	0/5 (0%)	0/5 (0%)	4/5 (80%)	2/5 (40%)	2/5 (40%)	1/5 (20%)	2/5 (40%)	2/5 (40%)	0/5 (0%)
Control	3/4 (75%)	0/4 (0%)	0/4 (0%)	1/4 (25%)	0/4 (0%)	3/4 (75%)	3/4 (75%)	3/4 (75%)	2/4 (50%)	0/4 (0%)	1/4 (25%)	0/4 (0%)	0/4 (0%)

Table 2Microscopic findings in each group of rabbits and organs.

**Table:** 

	**Lung**							**Heart**						
	**Bubble-like cavities**	**Congestion**	**Edema**	**Emphysema**	**Hemorrhages**	**Pneumonia**	**Thrombi**	**Bubble-like cavities**	**Congestion**	**Contraction band necrosis**	**Edema**	**Hemorrhages**	**Hyper-eosinophilia**	**Vacuolization**
C/D mortality	3/8 (38%)	7/8 (88%)	0/8 (0%)	8/8 (100%)	4/8 (50%)	1/8 (13%)	0/8 (0%)	2/8 (25%)	7/8 (88%)	1/8 (13%)	3/8 (38%)	4/8 (50%)	7/8 (88%)	7/8 (88%)
C/D euthanized	0/5 (0%)	3/5 (60%)	0/5 (0%)	5/5 (100%)	1/5 (20%)	0/5 (0%)	0/5 (0%)	0/5 (0%)	2/5 (40%)	0/5 (0%)	1/5 (20%)	2/5 (40%)	5/5 (100%)	5/5 (100%)
Control	0/4 (0%)	3/4 (75%)	0/4 (0%)	0/4 (0%)	0/4 (0%)	1/4 (25%)	0/4 (0%)	0/4 (0%)	1/4 (25%)	0/4 (0%)	0/4 (0%)	0/4 (0%)	2/4 (50%)	2/4 (50%)

**Table:** 

	**Liver**		**Kidney**		**Spleen**		**Brain**		**Skeletal muscle**		
	**Congestion**	**Vacuolization**	**Bubble-like cavities**	**Congestion**	**Congestion**	**Emphysema**	**Congestion**	**Hemorrhages**	**Edema**	**Hypereosinophilia**	**Wavy fibers**
C/D mortality	5/8 (63%)	4/8 (50%)	2/8 (25%)	8/8 (100%)	7/8 (88%)	6/8 (75%)	4/8 (50%)	2/8 (25%)	4/8 (50%)	7/8 (88%)	1/8 (13%)
C/D euthanized	5/5 (100%)	3/5 (60%)	3/5 (60%)	5/5 (100%)	5/5 (100%)	4/5 (80%)	2/5 (40%)	0/5 (0%)	0/5 (0%)	2/5 (40%)	2/5 (40%)
Control	1/4 (25%)	1/4 (25%)	0/4 (0%)	3/4 (75%)	3/4 (75%)	0/4 (0%)	0/4 (0%)	0/4 (0%)	0/4 (0%)	0/4 (0%)	0/4 (0%)

Pulmonary and splenic emphysema was observed in both groups. Emphysema in the adipose tissue was only seen in the C/D mortality group. Hemorrhages were more prevalent in different organs of the C/D mortality group, with hemorrhages present in the lung (50%), heart (50%), and brain (25%). The C/D euthanized group presented lower hemorrhages in the lung (20%) and heart (40%). No hemorrhages were seen in the brain.

Other relevant lesions observed were interfibrillar edema in cardiac (38% in C/D mortality and 20% in C/D euthanized) and skeletal muscles (50% in C/D mortality group) as well as hypereosinophilia in these fibers (88% in C/D mortality group and 40% in C/D euthanized group). Congestion was seen in most of the organs in all groups.

## Discussion

The main pathological finding in rabbits that died by experimental compression/decompression was the presence of a large amount of gas bubbles widely distributed throughout both the central and peripheral venous circulation. Emphysema (mainly in the lung, spleen, and adipose tissue) and hemorrhages in the lung, heart, and brain were the second main gross and histological finding in those rabbits. These pathological findings have also been described in beaked whales (Family *Ziphiidae*) and Risso's dolphins (*Grampus griseus*) that died of decompression-like sickness (7–12) (Figure 3D). Nevertheless, these lesions were absent in the rabbits that survived for 1 h after decompression.

The abundance, distribution, and gas composition of the animals from this study have been previously described in detail by Bernaldo de Quirós et al. (21). Thus, we will only briefly summarize those results here in order to compare them with the results from cetaceans. Macroscopic gas was observed massively and systematically distributed in rabbits that died due to a compression/decompression protocol. These results are in agreement with other studies such as those of Eggleton et al. (14), Lever et al. (16), or Shim et al. (17), carried out with guinea pigs, mice, and rabbits, respectively. In stranded cetaceans, the abundance and distribution of macroscopic gas were also analyzed in the two stranded Risso's dolphins diagnosed with a decompression-like sickness as the cause of death, being also systemically distributed (12) (Figures 2D,F).

Microscopic bubble-like cavities (i.e., small round to oval non-staining spaces that sometimes displaced erythrocytes) were observed within blood capillaries and small vessels from the lung, heart, and kidneys of the rabbits that died after decompression as well as in the kidney of animals that survived decompression and were euthanized. Microscopic gas embolism in the lung small pulmonary arteries, capillaries, and veins has been previously described, such as that of Geng et al. (4) in decompressed rabbits. L'Abbate et al. (24) also described microscopic bubbles in hepatic sinusoids from rats. In cetaceans diagnosed with a decompression-like sickness as the cause of death, abundant microscopic gas embolism was observed in renal capillaries, subcapsular veins, hepatic sinusoids, and pulmonary (Figure 3B), coronary, intestinal, and meningeal vessels (7, 10, 12).

These microscopic lesions were also observed in cetaceans, disrupting the white matter structure of the brain and spinal cord (7, 8). Similarly, micro-bubbles in the nervous system have been described in human medicine, being primarily seen in the spinal cord (25). It has been hypothesized that the low vascular supply and the high lipid content of the spinal white matter, conferred by the myelin that covers the axons, increase the affinity of inert gases for this structure (26). Thus, most CNS lesions are described in the spinal cord's white matter, such as punctured hemorrhages, spongiosis, axon swelling, and myelin degeneration (27). Microscopic bubble-like cavities were not observed in the brain or cranial spinal cord of the rabbits, but only the cranial part of the spinal cord was sampled. Future studies should aim at investigating the entire spinal cord.

Gas composition analysis of the gas embolism was performed in the rabbits of this study (21) and in some of the cetaceans diagnosed with a decompression-like sickness. These included a beaked whale stranded in association with naval exercises (11) and Risso's dolphins after a deadly prey interaction (12). In all cases, nitrogen was the main compound, followed by CO_2_. Hydrogen, a putrefaction marker, was absent or present in low quantities.

The most affected organ in all the rabbits from the C/D model was the lung. Mild to severe pulmonary emphysema was observed in all of them, while no control rabbits showed pulmonary emphysema. Similar results have been observed in other animal models of decompressive sickness with rats and rabbits (4, 19, 20, 28) as well as in mass stranded beaked whales associated with military exercises and in single stranded Risso's dolphins analyzed in this comparative study (7, 12) (Figure 2B).

Emphysema in other locations, such as the spleen and the adipose tissue of the abdominal cavity and mesenteric areas, was also observed in rabbits. Although splenic emphysema was observed to be affecting both groups in the C/D model, the severity was more critical in the rabbits that died after decompression, with severe emphysematous spleens vs. the mild emphysema of the spleens of rabbits that survived decompression and were euthanized. Clay (15) also described that half of the dogs analyzed presented macroscopic and microscopic gas in the spleen, which sometimes displaced the splenic follicles.

The adipose tissue (i.e., mesenteric, abdominal, and coronary fat depots) presented mild multifocal emphysema in most rabbits that died after decompression. Since nitrogen is more soluble in fatty tissues than in non-fat tissues (17), the relevant presence of bubbles within the adipose tissue in animals that have died by DCS was probably a macroscopic finding to be considered in the assessment of this disease. In the case of the cetaceans diagnosed with DCS, emphysema in the adipose tissue was evident in most cases, being more evident in the coronary fatty deposits and beneath the renal capsule (7, 12).

Another relevant injury found in the rabbits that died after decompression was hemorrhages in different organs. Bubbles can cause vasoconstriction, leading to the presence of ischemia, edema, and hemorrhages in target organs such as the lung (3). Severe pulmonary hemorrhages were present in the rabbits that died from compression–decompression than those that survived decompression after 1 h. Pulmonary hemorrhages have also been described in rats (19, 29), rabbits (4), and stranded cetaceans with pathological signs of DCS (7, 12).

The beaked whales in the mass strandings were all diagnosed with decompressive-like sickness (7, 8) that showed macroscopically acute disseminated hemorrhages in different organs, being especially severe in the CNS. These multifocal hemorrhages were mainly in subarachnoid areas, spinal cord, and meninges (Figure 3F). These findings are similar to those presented in rabbits from this study. In addition to hemorrhages in the CNS, vascular congestion, myelin degeneration, axon swelling, and pericapillary edema are common findings in pigs, humans, or rats (19, 26, 27, 30). However, in this experimental model, only congestion and brain-associated hemorrhages in the subarachnoid space were observed.

Interstitial and alveolar pulmonary edema was observed in all groups. This edema has also been macroscopically described in other experimental models that reproduced DCS in rats, sheep, and rabbits (4, 20, 28, 31). In these models, pulmonary edema was one of the most observed lesions, along with emphysema. According to Atkins et al. (31), pulmonary edema is related to the development of pulmonary hypertension and increased permeability of blood capillaries due to the contact of microbubbles with the endothelium, inducing the release of intracellular calcium, causing damage to endothelial cells, increasing their permeability, and allowing the release of protein-rich fluid into the intracellular space (20). In this experimental model, the low number of affected rabbits and all groups' presence do not seem relevant to this finding. Cetaceans diagnosed with DCS also had diffuse pulmonary edema (8) and non-specific lesions linked to different causes of death.

Acute muscle changes were associated with ischemic damage caused by stressful situations. In this study, these changes were found in muscular tissues such as skeletal muscle and myocardium. These acute changes usually occur within minutes after ischemia, including contraction band necrosis and wavy fibers' presence. These changes are well studied in other recent studies that analyzed the stress to which cetaceans were exposed while stranding alive (32) (Figure 3C). In this study, these two lesions were reported in fewer animals than expected (1/8 animals in the mortality group presented both lesions and 2/5 animals in the euthanized group presented wavy fibers). Other acute changes such as hypereosinophilia and intracytoplasmic vacuolization were observed in the cardiac and skeletal muscle of animals that died after decompression and, to a lesser extent, in those that survived and were euthanized. Since in the rabbits all the procedures were carried out under surgical anesthesia, this might prevent the appearance of some stress-related lesions.

Vacuolization of hepatocytes was observed in the C/D model, with the rabbits that died by the protocol being more affected. L'Abbate et al. (24) conducted a study on the changes observed in rats' liver after undergoing a rapid decompression protocol. Thus, hepatocellular vacuolization was not observed in spontaneous death or in the group euthanized after 3 h, but it was observed in the animals euthanized at 24 h, with different severity levels. These findings are dissimilar to those obtained in our study, where the animals that died shortly after decompression had more marked hepatocellular vacuolization than those that were euthanized at 1 h post-decompression.

While in this study no fibrin microthrombi were observed in the compression/decompression model, pulmonary arterial microthrombi have been described in other studies of DCS with similar protocols, such as in the study of Tanoue et al. (33), where rabbits were exposed to a compression protocol of 6 ATA for 40 min and rapid decompression of 5 min, and the animals euthanized immediately after decompression showed these microthrombi in large arteries of the lung, or in the study of Geng et al. (4), where thrombosis was seen in small pulmonary arteries, capillaries, and veins (7'98 ATA for 1 h, rapid decompression for 5 min in rabbits). Arieli et al. (18), with a rat model subjected to 12'49 ATA for 33 min and a rapid decompression in 6 min, also described the blood alterations generated by the microbubbles and the platelets' consequent activation which increased the presence of microthrombi and disseminated intravascular coagulation. In cetaceans, the presence of these microthrombi associated with decompression-like sickness was not observed.

In summary, it is necessary to highlight the difference between the severe presence of systemic gas embolism and associated gas lesions in rabbits dead by decompression vs. the absence or lower incidence in euthanized animals. Other studies have observed that, despite exposing individuals with a similar profile (species, sex, age, and weight) to the same protocol, bubble formation and lethality are highly variable (17, 24). Based on this, cetaceans exposed to the same diving profile and subjected to the same stress can present different results, with some animals developing a lethal DCS, while others may survive.

In conclusion, the rabbits that died after decompression presented large quantities of macroscopic and microscopic gas bubbles systemically distributed, emphysema, and hemorrhages in multiple vital organs. Most of the lesions described were probably due to the bubbles' mechanical and embolic damage. These same lesions have been described in cetaceans, consistent with a decompression-like sickness, reinforcing the pathological findings found. Besides this, almost half of the rabbits that survived for 1 h after decompression did not show the same lesions or severity. It reveals that individuality plays an essential role in this disease as it has previously been hypothesized in animal models and human diving medicine.

## Data Availability Statement

The original contributions presented in the study are included in the article/supplementary material, further inquiries can be directed to the corresponding authors.

## Ethics Statement

The animal study was reviewed and approved by Norwegian Committee for Animal Experiments (2154) and the Ethical Committee for Animal Experiments of the University of las Palmas de Gran Canaria (CEEBA-HUGCDN 002/2010).

## Author Contributions

AF, AM, and YB took charge of conceptualization. AF and AM took charge of funding. AV-W contributed to writing. YB, MC, AM, MA, and AV-W contributed to the experimental procedures and laboratory analyses. AV-W, YB, MC, and MA took charge of the pathological studies. All the authors contributed to review and editing. AF, YB, and MC supervised the study.

## Conflict of Interest

The authors declare that the research was conducted in the absence of any commercial or financial relationships that could be construed as a potential conflict of interest.
